# Supplementary material for: CryoEM reveals oligomeric isomers of a multienzyme complex and assembly mechanics
Source: J Struct Biol X. 2023 Apr 8;7:100088. doi: 10.1016/j.yjsbx.2023.100088 (PMC10148081; doi:10.1016/j.yjsbx.2023.100088)
Supplement: Supplementary data 2 [file mmc2.docx]

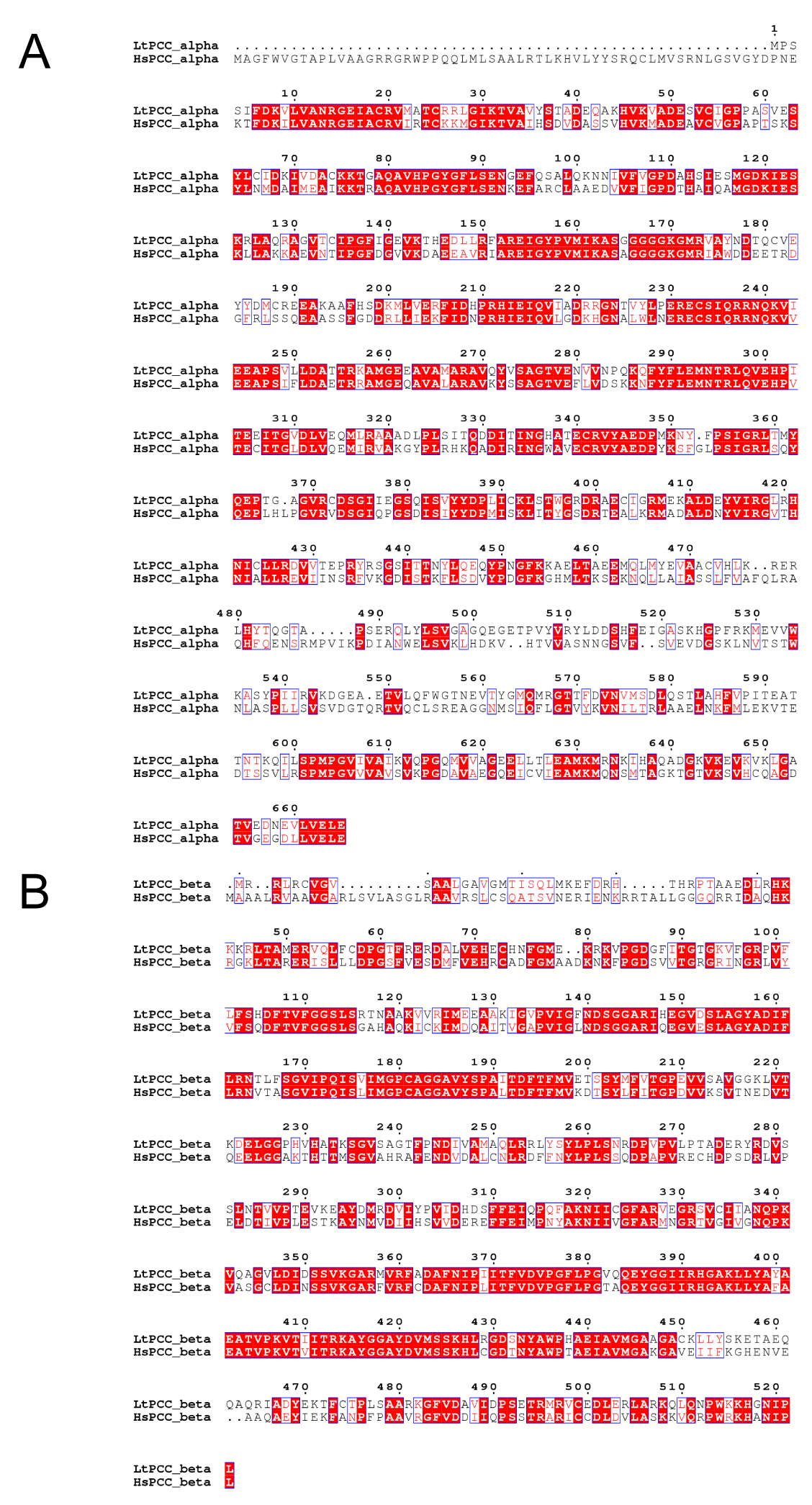


Fig. S1. Sequence alignment of LtPCC α-subunits (A) and β-subunits (B) with HsPCC α-subunits and β-subunits. There is a dot on top of every ten residues. Blue-outlined boxes denote similar residues. Red background denotes identical residues.

**
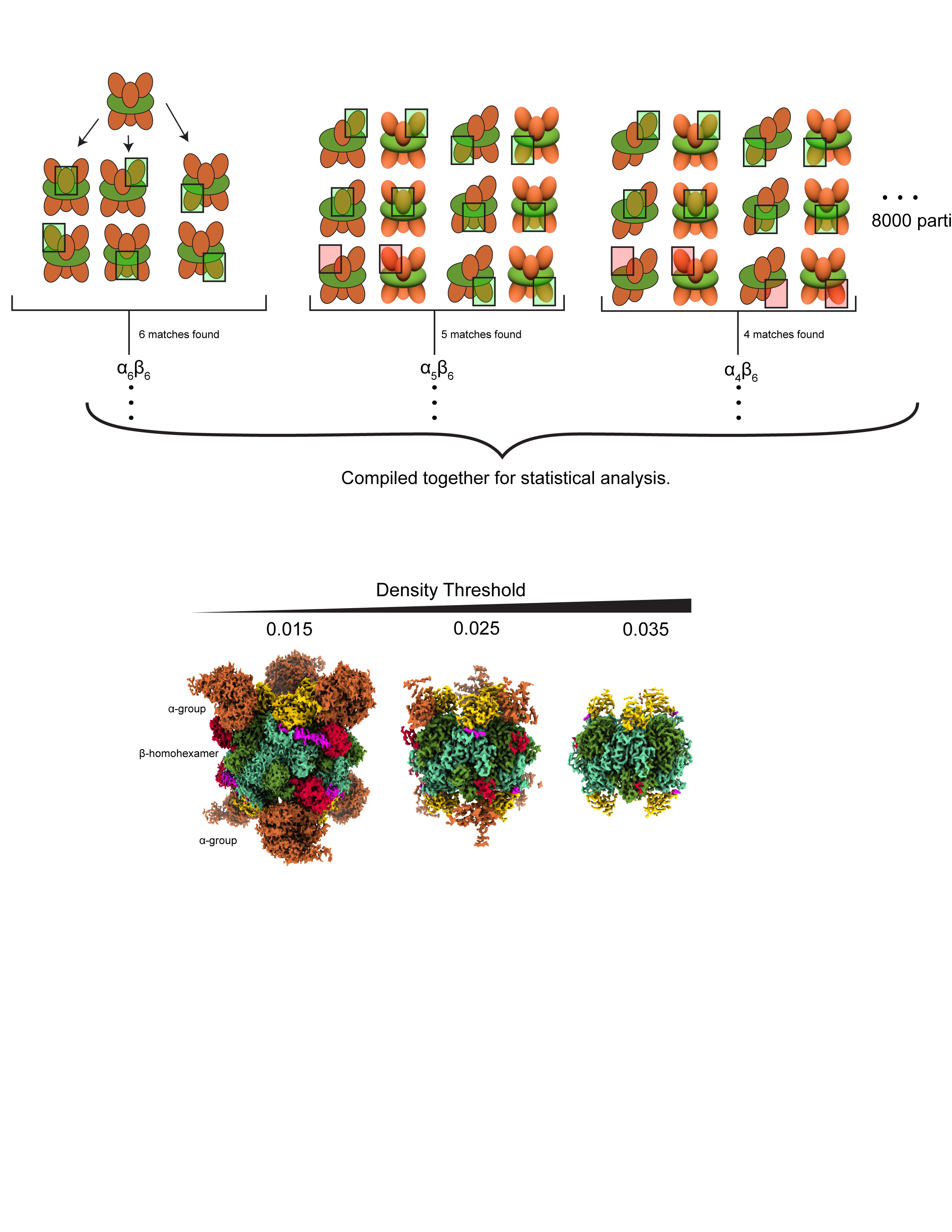
**

Fig. S2. In D3 reconstruction, LtPCC α-subunits exhibit flexibility and/or lower occupancy at high threshold while the β-homohexamer remains stable.


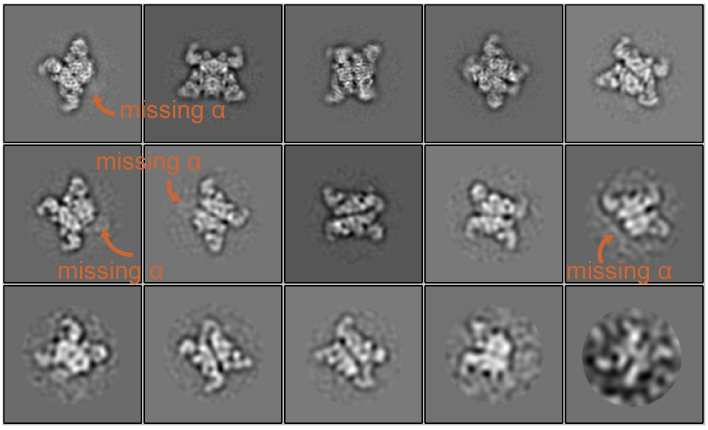


Fig. S3. All selected 2D classes for LtPCC reconstruction with D3 symmetry.


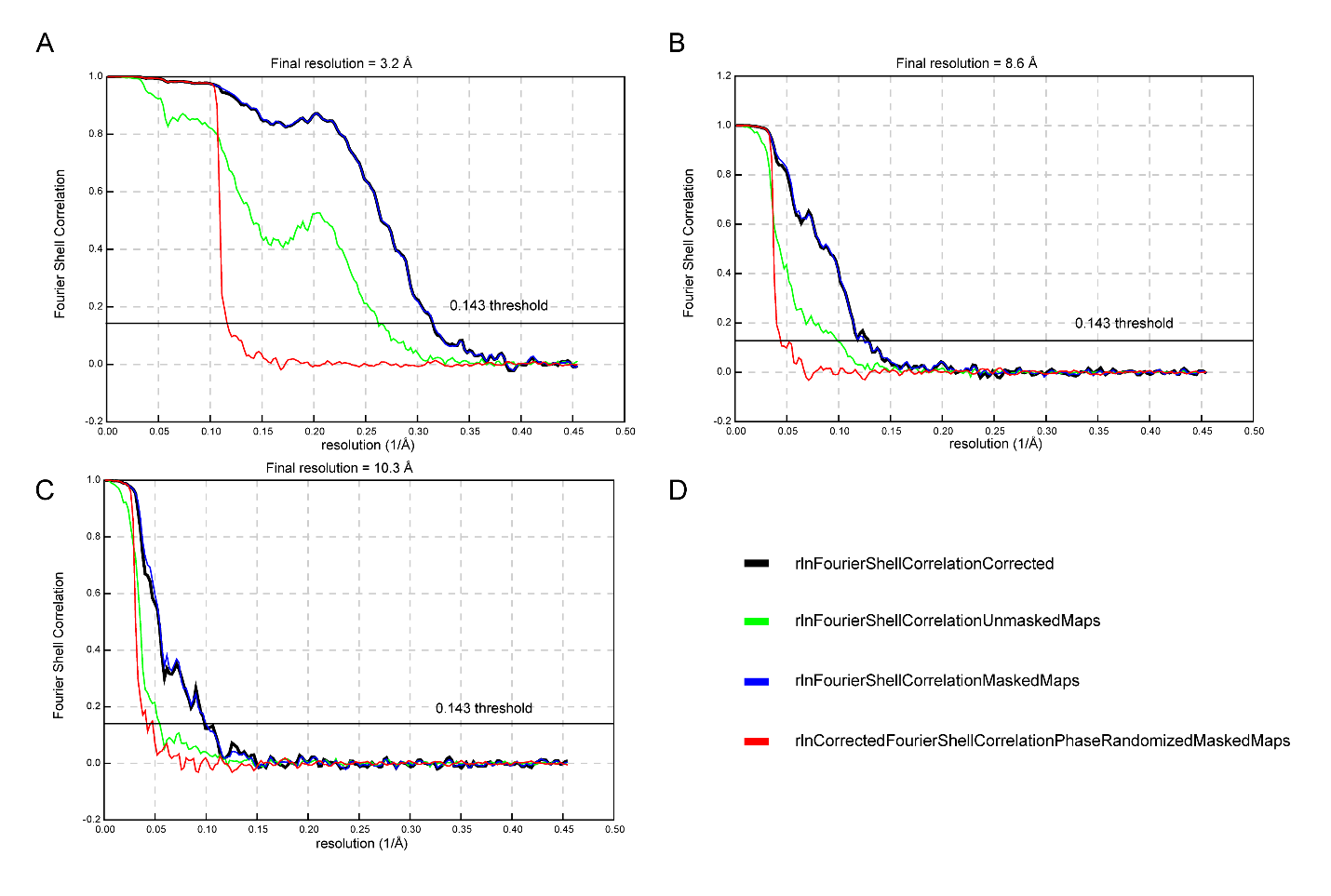


Fig. S4. Estimation of resolution for the three cryoEM maps. Fourier shell correlation curves as colored in (D) for the α_6_β_6_ map (A) α_5_β_6_ map (B) α_4_β_6_ map (C).


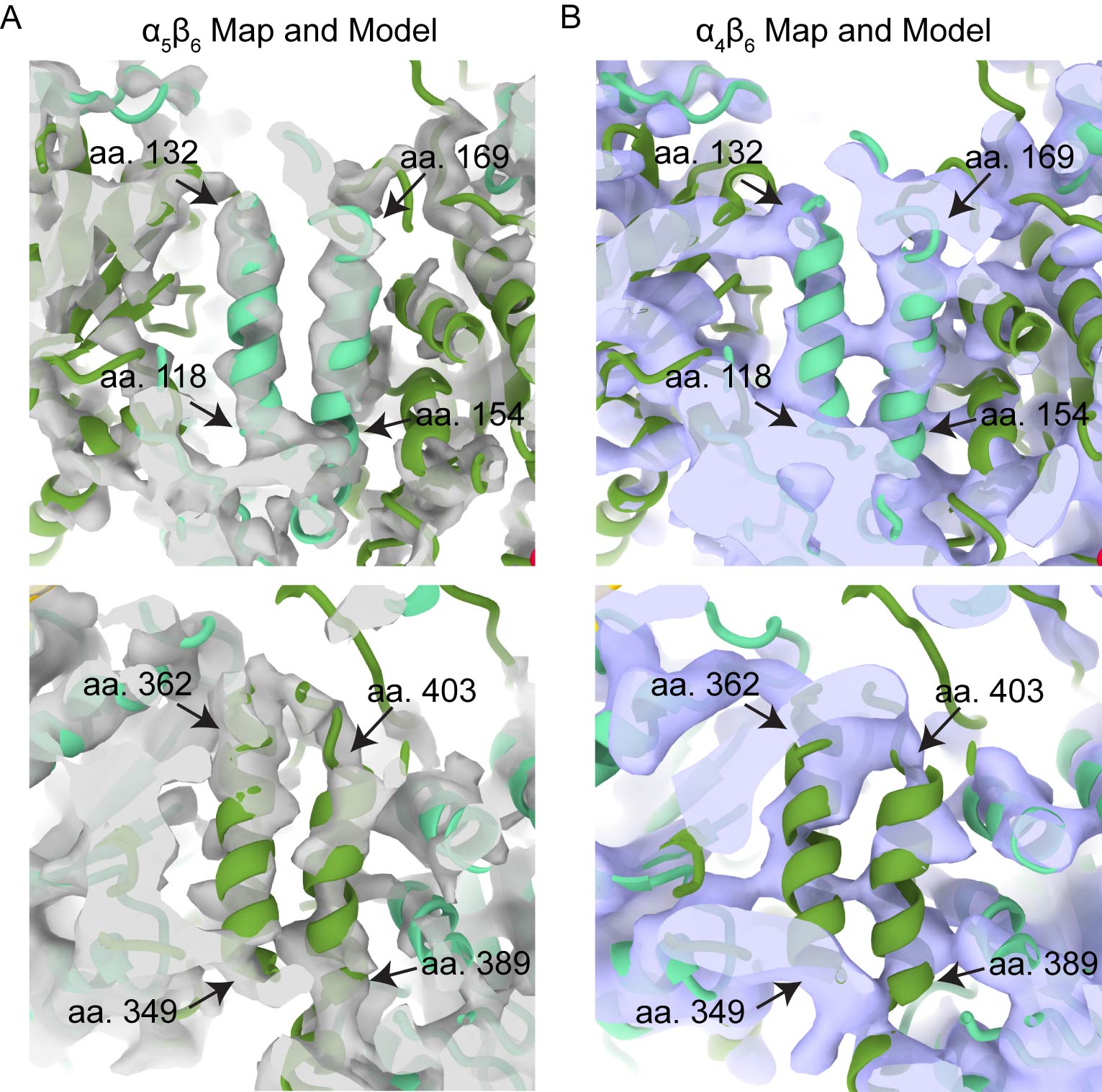


Fig. S5. Selected densities for the (A) α_5_β_6_ map (B) α_4_β_6_ map, showing secondary structure.
